# Image improvement of temporal focusing multiphoton microscopy via superior spatial modulation excitation and Hilbert–Huang transform decomposition

**DOI:** 10.1038/s41598-022-14367-8

**Published:** 2022-06-16

**Authors:** Yvonne Yuling Hu, Chun-Yu Lin, Chia-Yuan Chang, Yuan-Long Lo, Shean-Jen Chen

**Affiliations:** 1 Department of Photonics, National Cheng Kung University, Tainan, 70101 Taiwan; 2 College of Photonics, National Yang Ming Chiao Tung University, Tainan, 71150 Taiwan; 3 Department of Mechanical Engineering, National Cheng Kung University, Tainan, 70101 Taiwan; 4 Department of Engineering Science, National Cheng Kung University, Tainan, 70101 Taiwan; 5 Taiwan Instrument Research Institute, National Applied Research Laboratories, Hsinchu, 300 Taiwan

**Keywords:** Engineering, Optics and photonics

## Abstract

Temporal focusing-based multiphoton excitation microscopy (TFMPEM) just provides the advantage of widefield optical sectioning ability with axial resolution of several micrometers. However, under the plane excitation, the photons emitted from the molecules in turbid tissues undergo scattering, resulting in complicated background noise and an impaired widefield image quality. Accordingly, this study constructs a general and comprehensive numerical model of TFMPEM utilizing Fourier optics and performs simulations to determine the superior spatial frequency and orientation of the structured pattern which maximize the axial excitation confinement. It is shown experimentally that the optimized pattern minimizes the intensity of the out-of-focus signal, and hence improves the quality of the image reconstructed using the Hilbert transform (HT). However, the square-like reflection components on digital micromirror device leads to pattern residuals in the demodulated image when applying high spatial frequency of structured pattern. Accordingly, the HT is replaced with Hilbert–Huang transform (HHT) in order to sift out the low-frequency background noise and pattern residuals in the demodulation process. The experimental results obtained using a kidney tissue sample show that the HHT yields a significant improvement in the TFMPEM image quality.

## Introduction

Multiphoton excitation (MPE) microscopy is a well-established technique for in vivo biological imaging. By applying an ultrashort pulse laser and an objective lens with a high numerical aperture (NA), the photons emitted from the sample molecules are densely collected in a submicron volume at the front focal point of the objective lens, and induce a fluorescence signal with an intrinsic optical sectioning ability^1^. MPE system utilizes a near infrared (NIR) wavelength as excitation source due to the lower absorption and scattering effects, which extend the penetration depth and reduce the photobleaching and phototoxicity phenomena in biotissue^2^. Through the use of three-axis point scanners, such as galvanometers and polygon scanners^3^, MPE microscopy provides three-dimensional (3D) images with an extremely high spatial resolution, and is hence a powerful tool for many fields of biological research, including neurobiology, angiology, embryology, and dermatology^4–7^. However, the point-by-point scanning methods restrict the available frame rate, and therefore compromise the feasibility of MPE for dynamic detection application, such as neural signaling^8^.

In contrast to point scanning MPE, temporal focusing-based multiphoton excitation microscopy (TFMPEM) provides a wide field-of-view, and hence enhances the temporal resolution^9^. Briefly, a diffraction component (e.g., a blazed grating) is used to spectrally disperse the laser pulse into different angles in accordance with the diffraction equation. The decomposed light is passed through a 4f imaging system consisting of a collimating lens and an objective lens, and then overlaps in phase at the front focal plane of the objective lens to achieve temporal focusing^10–12^. The broadened pulse is finally reconstructed at the temporal focusing plane to form the shortest pulsed width possible and provide a sufficient photon density to generate a two-photon (2P) excitation effect. Notable, TFMPEM makes possible an optical sectioning ability with an axial excitation confinement (AEC) of just several micrometers given an appropriate setting of the system parameters, such as the laser pulse width, initial beam size, diffraction groove density, and objective lens NA^13–15^.

TFMPEM has been successfully used for many imaging applications including rapid 3D Brownian motion tracking^16^, widefield fluorescence lifetime imaging^17^, and in vivo 3D neuronal activity analysis^18^. TFMPEM has also been applied to trap small objects in 3D space using holographic optical tweezer^19^ and to perform micromachining via multiphoton-induced ablation with the assistance of greyscale patters and optical mask^20,21^. In addition, TFMPEM has been used in conjunction with spatial light modulator (SLMs) and digital micromirror device (DMD) to produce structured light for pulse-shaping and patterned illumination application^22,23^.

Structured illumination is commonly used in widefield optical microscopy to perform optical sectioning^24^, eliminate background noise^25^, and provide super-resolution^26^. In TFMPEM scheme, SLM- or DMD-generated patterns provide an effective means of expanding the detected spatial frequency and suppressing the diffraction limit in the lateral and axial dimensions^27,28^. Furthermore, modulation of the pattern frequency enhances the effective NA of the objective lens by filling the back aperture, and hence improves the AEC. Finally, suitably-designed reconstruction methods enable the efficient collection of the higher spatial frequency components of the modulated light pattern and the rejection of the out-of-focus noise^29–31^.

DMD is one of the most economic and adaptable devices for producing structured light in TFMPEM scheme. A DMD is composed of a two-dimensional array of diagonal micromirrors, where each mirror, which acts as an independent pixel, can either reflect or block incoming light by tilting through a ± 12 angle relative to the midline^32^. Given the fixed flipping angle of the micromirrors, the DMD functions as a blazed grating when irradiated by a pulsed laser beam with a proper angle, and thus introduces angular dispersion and spectral separation of the beam in accordance with the diffraction equation. However, through an appropriate control of the individual mirrors, the DMD can also generate arbitrary lighting patterns and act as an optical mask. DMD-based TFMPEM system has been successfully applied to improve the image quality through the use of nonlinear structured-illumination techniques^33^, HiLo reconstruction^34^, and Hilbert transform (HT)-based demodulation^35^. HiLo reconstruction uses two raw images, one with uniform illumination and the other with patterned illumination, to reject the background noise and hence improve the reconstructed image quality. However, the HiLo filtering parameters require case-by-case manual fine tuning, which limits the application of the reconstruction process for bulk image acquisition. The HT demodulation process uses two patterned images with the same spatial frequency but a 180° difference in phase. Moreover, the Hilbert–Huang transform (HHT) shows more adaptive than HT when applying in a nonlinear scheme such as 2P phenomena.

In theory, a spatial frequency close to the diffraction limit enhances the AEC in TFMPEM and improves the image quality. However, for high spatial frequency patters, any inaccuracies in the phase difference produced by the DMD are dramatically magnified by the nonlinear imaging mechanism. As a result, pattern residuals often remain in the reconstructed image following the demodulation process^36^. Accordingly, this study first performs numerical simulations based on an advanced Fourier optics model to determine the spatial frequency and orientation of the modulation signal which maximize the AEC effect in a DMD-based TFMPEM system. The HHT is then used to decompose the detected image into intrinsic mode functions (IMFs) and reconstruct the image without out-of-focus noise and pattern residuals under widefield 2P microscopy.

## Results

### Verification of Fourier optics model

**Figure 1:**
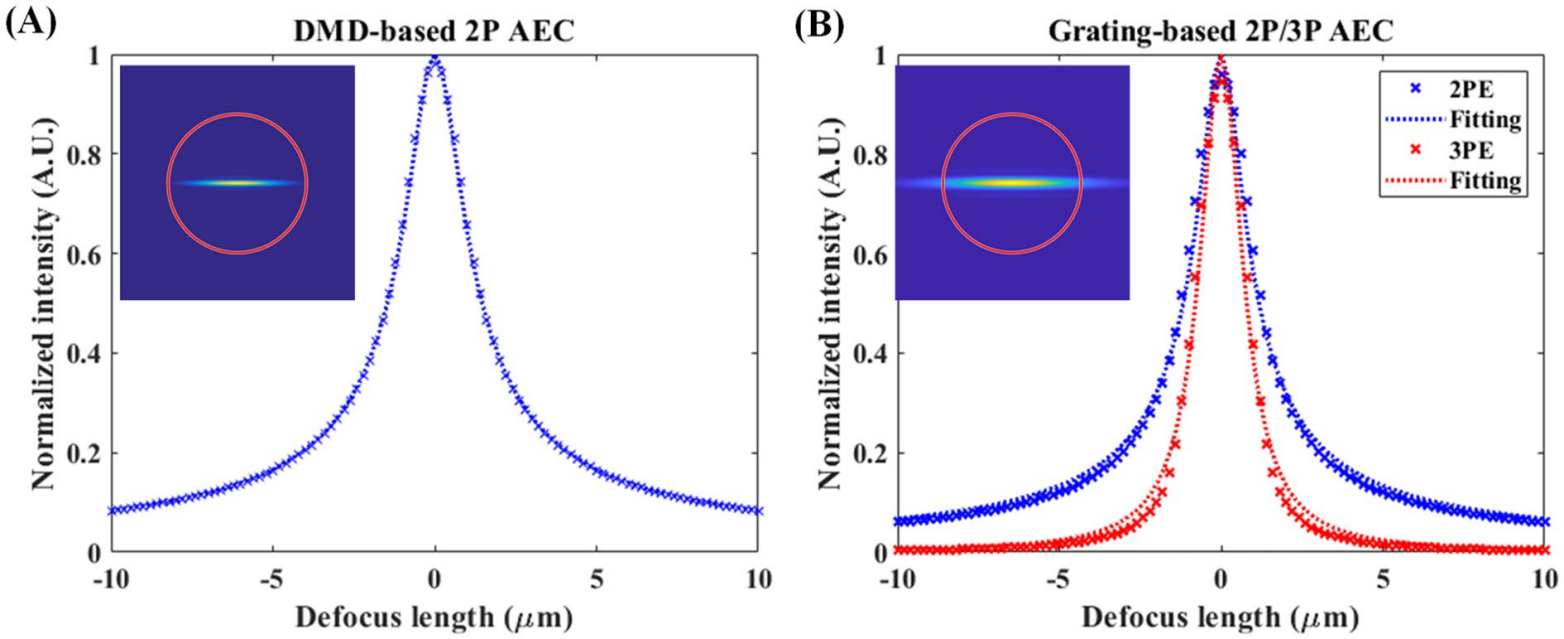
Simulated AEC of UPlanSApo 60XW objective lens in: (**A**) DMD-based 2P TFMPEM^32^ and (**B**) grating-based 2P and 3P TFMPEM^37^. For both figures, the inserts show the intensity distribution of the dispersed beam at the back focal plane of the objective lens. In addition, the red circles indicate the effective coverage of the back aperture, which is approximately 5.4 mm in diameter.

The validity of the proposed Fourier optics model was evaluated by comparing the simulation results for the AEC profile of two TFMPEM schemes (i.e., DMD-based and grating-based) with the corresponding results reported in the literature. For a DMD-based system^32^, the spectral profile of the ultrashort pulse was assumed to be Gaussian with a central wavelength of 800 nm and a full width at half maxima (FWHM) of approximately 7 nm. In addition, the diffraction efficiency of the DMD at tenth-order was equivalent to that produced by a 517 lines/mm blazed grating. The effective focal lengths of the collimating lens and objective lens (UPlanSApo 60XW, NA 1.2, Olympus, Japan) were 625 mm and 3 mm, respectively. Finally, the effective diameter of the back aperture of the objective lens was 5.4 mm. Figure 1A shows the simulation results obtained for the AEC of the TFMPEM system under 2P excitation. Based on the FWHM of the fitted intensity profile^12^, the AEC was calculated to be approximately 2.93 μm. The calculated value was thus in good agreement with the experimental results of 3.0 μm reported in the literature for a thin Rhodamine 6G film (< 200 nm)^32^.

The validity of the proposed numerical model was further investigated using the 2P and three-photon (3P) TFMPEM schemes, reported by Toda et al.^37^ The spectral profile of the ultrashort pulse was assumed to be a squared hyperbolic secant function (Sech^2^) with a central wavelength of 1060 nm and a FWHM of approximately 13 nm. The pulse width was compressed to 92 fs to enhance the excitation efficiency, and a blazed grating with a groove density of 830 lines/mm was used as the diffraction component. The system used the same objective lens as that considered in the previous case (UPlanSApo 60XW, NA 1.2, Olympus, Japan). However, the collimating lens had a shorter focal length of 375 mm. Figure 1B shows the simulation results obtained for the 2P and 3P AEC profiles of the TFMPEM system. The calculated 2P and 3P AEC values (i.e., 2.34 μm and 1.6 μm, respectively) were found to be in close agreement with the experimental values reported by Toda et al.^37^ (i.e., 2.1 μm and 1.58 μm, respectively). Overall, the good agreement between the numerical values of the AEC presented in Fig. 1A,B and the experimental values reported in the literature^32,36^ confirm the feasibility of the proposed numerical model.

### Modulation of spatial frequency and orientation

**Figure 2:**
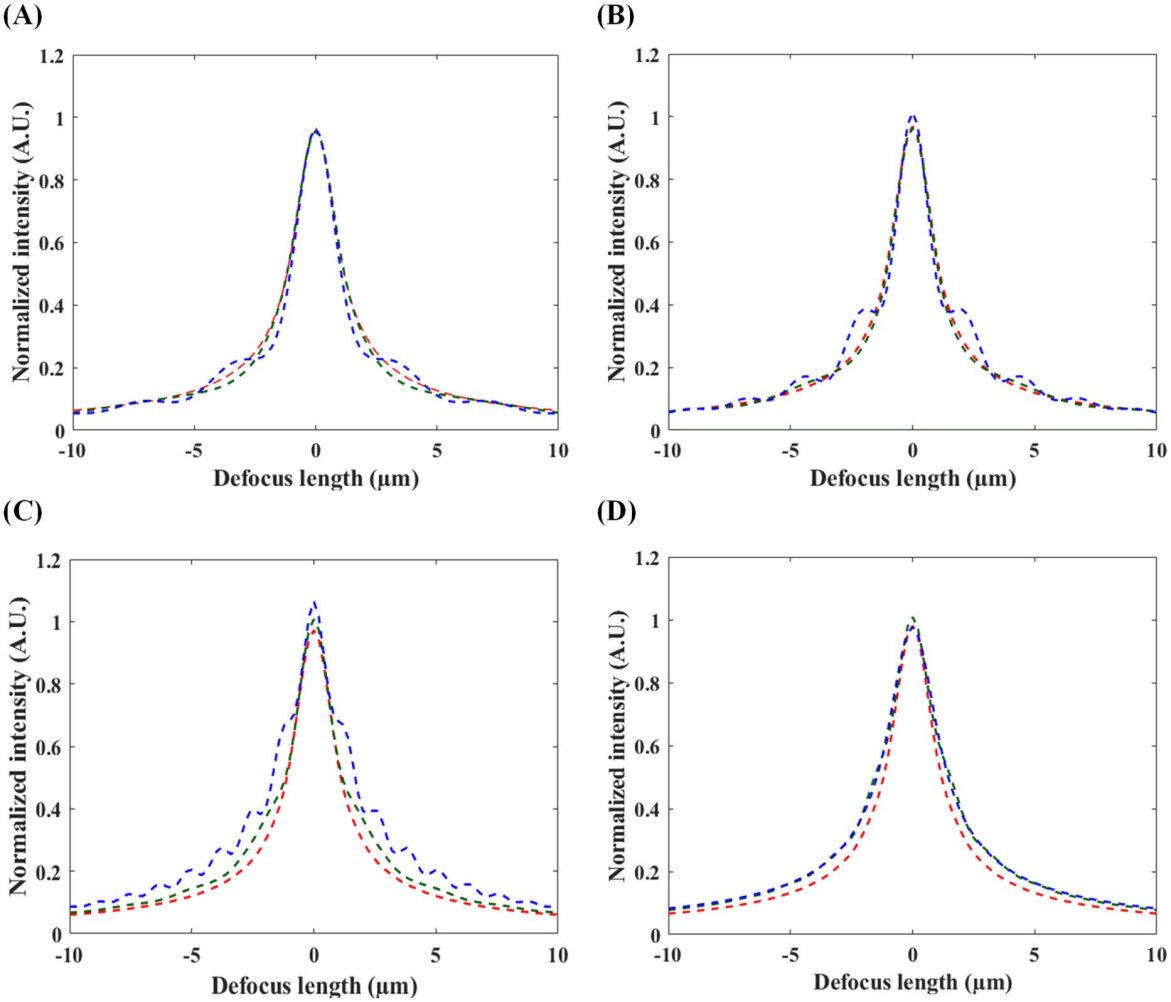
AEC profiles for four sinusoidal structured light patterns with spatial frequencies of: (**A**) 0.65 μm^−1^, (**B**) 0.81 μm^−1^, (**C**) 1.09 μm^−1^, and (**D**) 1.3 μm^−1^. Note that each figure shows three orientations of the modulated light pattern, i.e., 0° (red), 45° (green), and 90° (blue).

In general, the spatial frequency of the structured lighting pattern contributes significantly to the AEC in TFMPEM^30^. Figure 2A–D show the simulated AEC profiles of four sinusoidal light patterns modulated with spatial frequencies (*k*_*P*_) of 0.65 μm^−1^, 0.81 μm^−1^, 1.09 μm^−1^ and 1.3 μm^−1^, respectively, and three different orientation angles (0° (red), 45° (green), and 90° (blue)). Note that the TFMPEM scheme is assumed to utilize a water-immersion objective lens (UPlanSApo 60XW, NA 1.2, Olympus, Japan) with a restricted aperture and a diffraction-limit (cut-off) frequency of approximately 1.13 μm^−1^ (i.e., NA/(*λ* × *n*_water_) = 1.2/(0.8 μm × 1.33) = 1.13 μm^−1^). Moreover, an orientation angle of 0° indicates that the modulated pattern is aligned with the dispersion direction. It is seen that for the finest spatial frequency of 0.65 μm^−1^, the FWHM of the AEC profile is equal to approximately 2.15 μm for an orientation angle of 90° and 0.81 μm^−1^ for an orientation angle of 45° (see Fig. 2A). For a finer spatial frequency of 1.09 μm^−1^ (see Fig. 2C), the FWHM at 90° increases to 3.13 μm due to the presence of local peaks on the side lobes of the AEC profile. Moreover, a notable difference in the FWHM values is observed for the three different modulation orientations. However, as the spatial frequency is further increased to 1.30 μm^−1^, the FWHM of the AEC profile is insensitive to the modulation orientation (see Fig. 2D) due to the inherent restriction in the effective aperture at the back focal plane of the objective lens. The simulation results also show that the finer pattern induces Talbot effect. As the plane wave incidents into periodical structure, such as a blazed grating, the pattern of the structure will repeatedly occur when the wave propagates. In two-photon mechanism, when the pattern orientation is perpendicular to that of the grating, a Talbot image with phase shift of 180° will occur when the distance away from the grating is equal to:

$$z=\frac{\left(2n-1\right){L}^{2}}{\lambda },$$

where *L* is the period of the pattern, and *n* is positive integer. This near-field diffraction effect induces an inverse structural pattern and introduces side lobes that shown in Fig. 2A–C, which degrades the axial confinement. Overall, the simulation results confirm that the FWHM of the AEC profile can be tuned through an appropriate setting of the modulation parameters (i.e., the spatial frequency and orientation).

### Verification of AEC improvement

**Figure 3:**
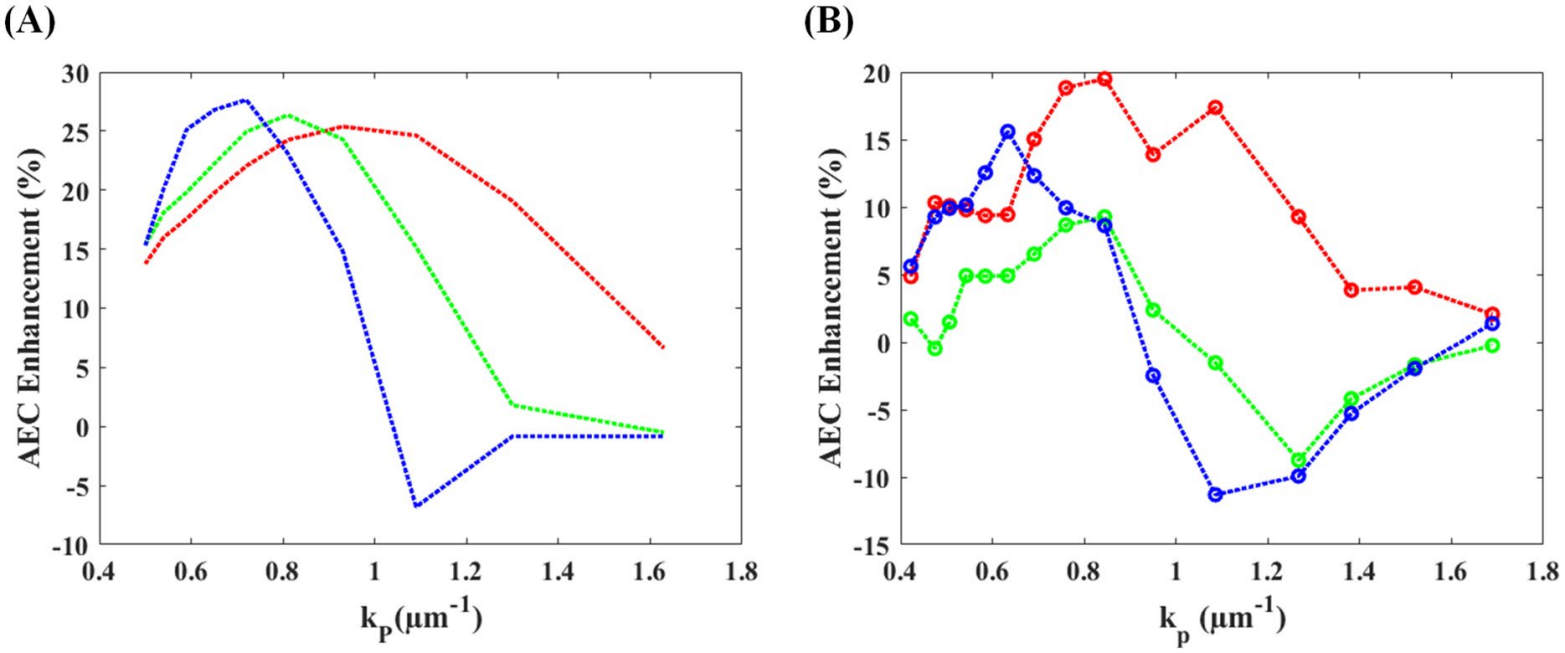
AEC enhancement of structured light patterns with different spatial frequencies and orientations: (**A**) simulation results and (**B**) experimental results. Note that each figure shows three orientations of the modulated light pattern, i.e., 0° (red), 45° (green), and 90° (blue).

In order to determine the superior design of the sinusoidal modulation pattern, the AEC profiles of the TFMPEM scheme reported by Chang et al.^30^ were evaluated both numerically and experimentally for spatial frequencies in the range of 0.5 to 1.63 μm^−1^ and modulation orientations of 0°, 45° and 90°, respectively. The confinement effect of the AEC profile under each of the considered modulation conditions was evaluated using the AEC enhancement metric.

$$\mathrm{AEC Improvement}= \left(1-\frac{{\mathrm{AEC}}_{\mathrm{with\, pattern}}}{{\mathrm{AEC}}_{\mathrm{uniform\, pattern}}}\right)\times 100\mathrm{\%},$$

where larger value indicates AEC with pattern illumination shows higher improvement than that of uniform pattern. Figure 3A,B show the simulation and experimental results obtained for the AEC improvement in every case. As shown in Fig. 3A, the FWHM values of the AEC profiles at orientation angles of 0° and 45° are generally smaller than those produced using uniform illumination. Moreover, the spatial frequencies associated with the maximal AEC improvement at modulation orientations of 0°, 45° and 90°, are 0.92 μm^−1^, 0.81 μm^−1^ and 0.65 μm^−1^, respectively. Similarly, the experimental results show that the maximal improvement in the AEC is obtained using spatial frequencies of 0.84 μm^−1^, 0.84 μm^−1^, and 0.63 μm^−1^ at modulation angles of 0°, 45°, and 90°, respectively. Both simulation and experimental results conform with previous work presented by Chang et al.^30^ In general, the simulation and experimental results for the superior spatial frequency are in good qualitative agreement with one another under each of the modulation orientations. However, the simulation results generally overstate the magnitude of the AEC enhancement compared to the experimental values. This finding suggests that the back aperture of the objective lens may block the extended spatial frequency, which inversely decrease the coverage of the spatial frequency.

### Image reconstruction results using biological sample

**Figure 4:**
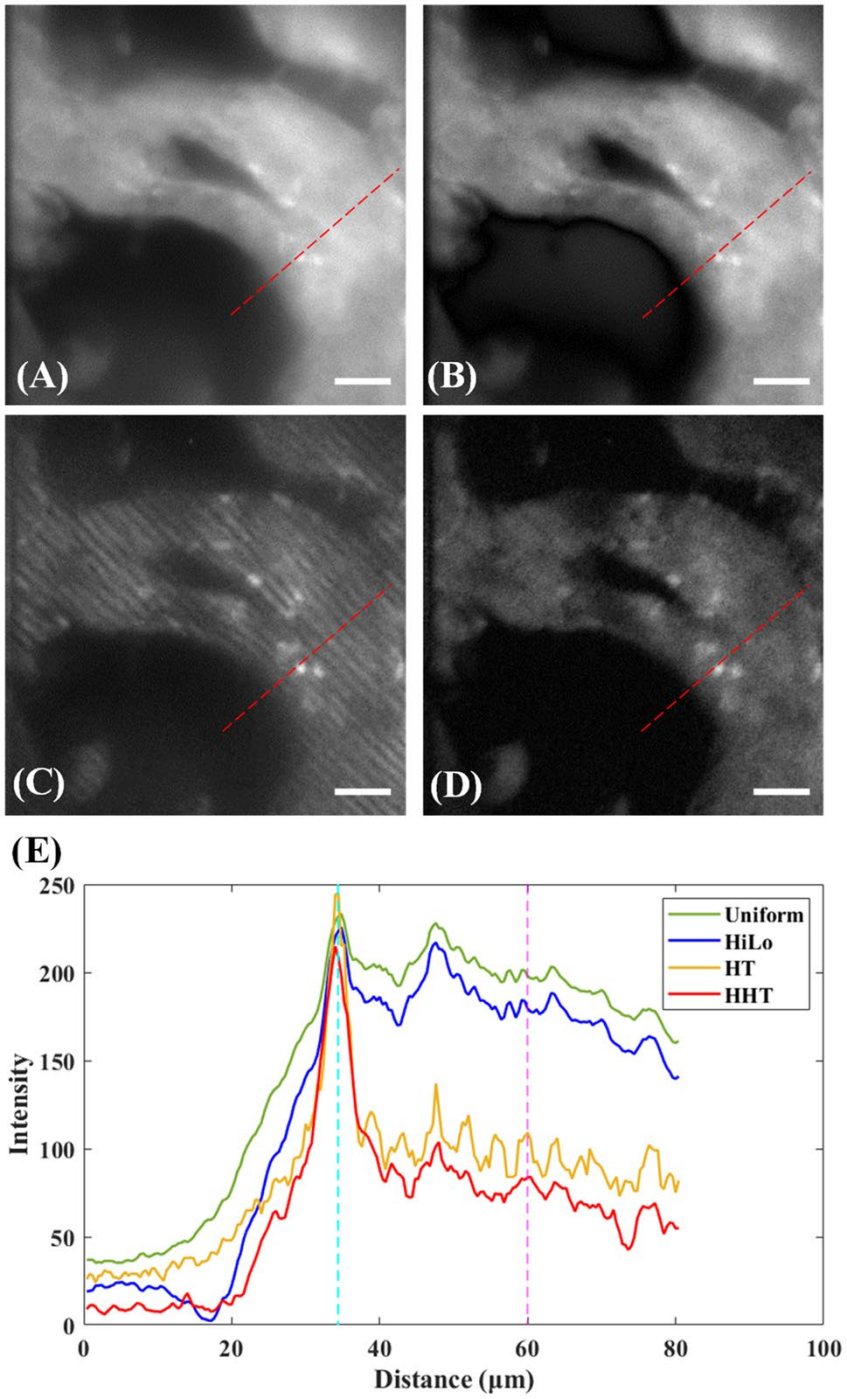
(**A**) Original TFMPEM image with uniform illumination, and reconstructed images obtained using: (**B**) HiLo, (**C**) Hilbert transform, and (**D**) Hilbert–Huang transform. (**E**) Intensity profile along red dashed line in each figure. Note that the scale bar indicates 20 μm in every case.

Based on the simulation and experimental results presented in Fig. 3, the superior spatial frequency of the modulated light pattern was determined to be 1.06 μm^−1^. A series of experimental imaging trials was performed using a 100-μm thick eGFP-expressed kidney slice as the sample. The kidney slice shows three features, including nucleus, tubular epithelium, and cavity. Figure 4A shows the TFMPEM image obtained under uniform illumination conditions, where most of the intensity of the detection signal is contributed by the out-of-focus region of the sample. Since the AEC profile is not well-confined, all of the fluorescence molecules within the focal volume have a high probability of being excited. Consequently, signal cross-talk occurs between layers, and hence the collected image contains not only the desired layer of tissue, but also the surrounding tissue information. As shown in Fig. 4B, the image contrast can be enhanced by using a structured lighting pattern and applying the HiLo reconstruction algorithm with appropriately-adjusted parameters. However, the cross-talk phenomenon still remains. Figure 4C shows the TFMPEM image obtained when using the HT to reconstruct the modulated signal. It is apparent that the background noise is significantly reduced. However, since the structured lighting pattern is formed by the multiple discrete pixel elements in the DMD, the use of a higher spatial frequency (*k*_*P*_ = 1.06 μm^−1^) results in a square-like signal, which leads to the generation of pattern residuals in the reconstructed image. Accordingly, the reconstruction process was also performed using the HHT for comparison purposes. The image presented in Fig. 4D shows that the HHT results in a significant reduction in the background signal and pattern residuals, while preserving the intensity of the in-focus structure. Figure 4E shows the intensity profiles along the red dashed line in each figure. As mentioned previously, HiLo reconstruction is manually tunable, which means the signal-to-noise ratio (SNR) is biased based on the tuned parameters and does not obeyed the law of energy conservation as well. On the contrary, HT and HHT only rely on the algorithm to reconstruct the image. To compare the background removal results, the features along the intensity profiles are pointed out for quantitively discussion. First, the contrast between the nucleus and the tubular epithelium, in which the distance along the profile are 26 µm and 60 µm, respectively. The contrast of the two points is 1.18, 1.26, 2.24, and 2.56 for uniform, HiLo, HT, and HHT images, respectively. The quantitate numbers show that the on-focus signal (i.e., the nucleus) is kept after the reconstruction, while most of the out-of-focus signal (i.e., part of the tubular epithelium) is well removed. The results confirm that the HHT image has the lowest out-of-focus signal intensity of the various images, while still preserving most of the on-focus information.

**Figure 5:**
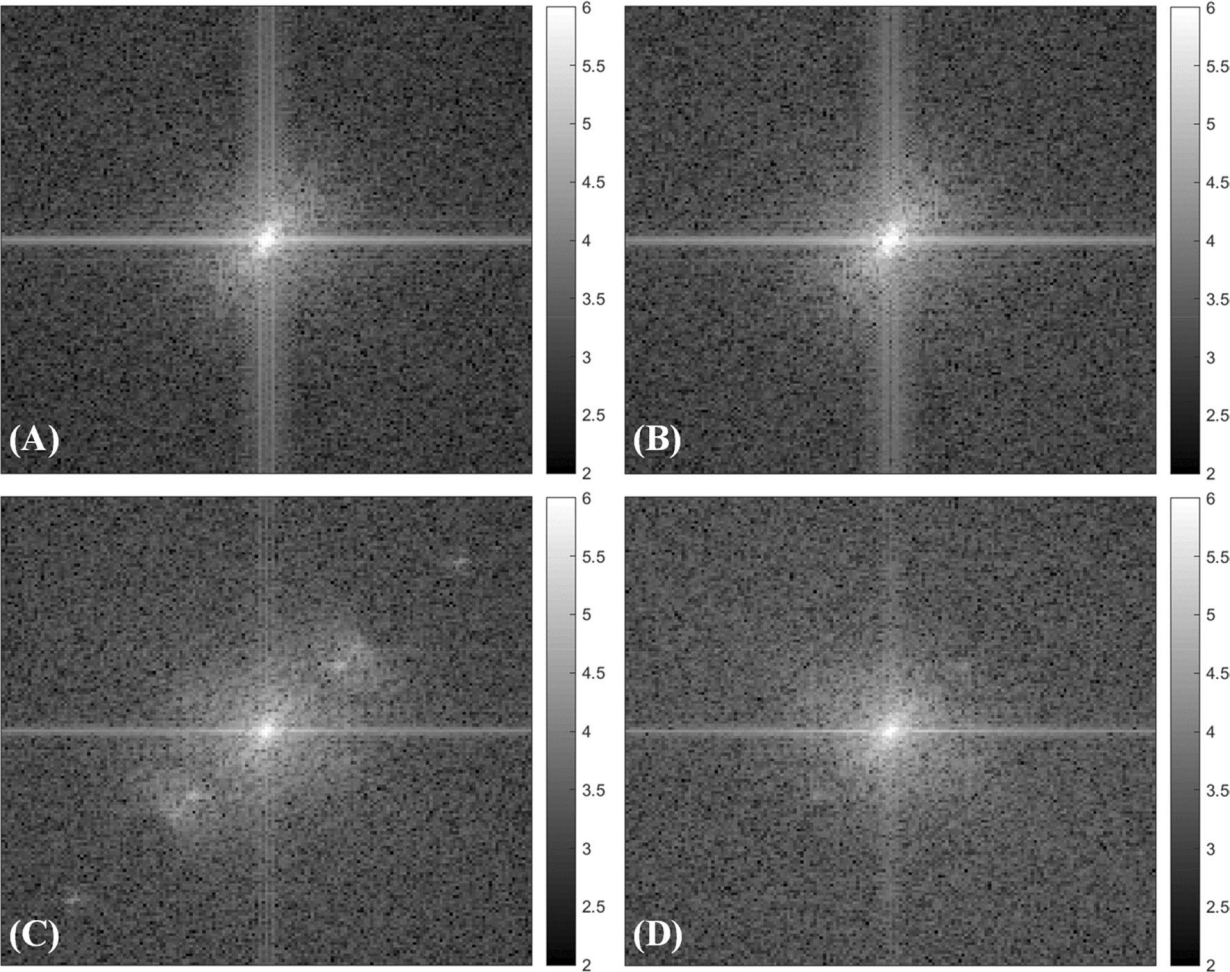
Power spectra of: (**A**) original TFMPEM image with uniform illumination, and reconstructed images obtained using: (**B**) HiLo, (**C**) Hilbert transform, and (**D**) Hilbert–Huang transform.

Figure 5 shows the power spectra of the four images in Fig. 4. It is seen that the power spectrum of the image reconstructed using the HHT (Fig. 5D) is similar to that of the original TFMPEM image (Fig. 5A) and HiLo reconstructed image (Fig. 5B). By contrast, the image reconstructed using the HT contains high-frequency residuals (Fig. 5C). Thus, the effectiveness of the HHT reconstruction method in eliminating the pattern residuals in the reconstructed image is confirmed.

## Discussion

DMD-based TFMPEM provides a wide excitation area, thereby enabling a higher frame rate and a lower possibility of photodamage. The image quality of TFMPEM systems can be enhanced by using the HiLo reconstruction method to block out the background signal in the demodulation process. However, the reconstruction process requires a case-by-case manual adjustment of the HiLo parameters (e.g., the filter size and the weighting). Furthermore, non-optimal settings of the HiLo parameters may result in aliasing of the reconstructed images. The HT overcomes this problem by using two patterned images with a phase difference of π between them to reconstruct the image. However, at high spatial frequencies, the detection signal becomes square-like and a nonlinear photon excitation effect is induced, which degrades the accuracy of the reconstruction process and produces pattern residuals in the reconstructed image. Accordingly, the present study provides a globally adaptable and comprehensive numerical simulation for pattern illumination under temporal focusing scheme and utilizes HHT to automatically perform image reconstruction instead. In particular, the HHT algorithm decomposes the detection signal into multiple IMFs in accordance with their frequencies and then processes these IMFs using the HT after first removing the IMFs corresponding to background noise. The reconstructed image not only contains fewer pattern residuals, but also has a lower out-of-focus signal (i.e., an improved optical sectioning performance). The TFMPEM system is thus applicable to various high-speed imaging applications, such as transient calcium signaling and motile cell tracking.

## Methods

### Configuration of experimental TFMPEM

The detailed configuration of the DMD-based TFMPEM employed in the present study is described in a previous study by the present group^30^. However, briefly, the DMD (DLP7000, Texas Instrument, USA) had a 0.7-inch illumination area and a 1,024 × 768 micromirror array with a pitch size of 13.68 μm and was orientated at an angle of 45° with respect to the incoming light. The structure of the DMD was equivalent to a blazed grating with a blazed angle of 12°. The central wavelength of the utilized laser, which is a Ti:Sapphire regenerative amplifier (Spitfire Pro XP, Spectra-Physics, USA) coupled with a Ti:Sapphire ultrafast oscillator (Tsunami, Spectra-Physics, USA) as seed beam, is at 800 nm and the bandwidth is 7 nm. The 10th-order diffraction of the ultrafast laser was adopted in order to approach the maximum power set through the diffraction equation. The dispersion efficiency of the DMD was verified as being equivalent to a 517 lines/mm blazed grating when using the 1st-order diffraction of the same wavelength. Through the use of a high definition multimedia interface (HDMI), the DMD served not only as a dispersion element for temporal focusing, but also a spatially-modulated illuminator with the ability to produce 8-bit patterns.

### Numerical simulation of patterned-illumination TFMPEM

The simulations conducted in the present study were based on an advanced numerical model of TFMPEM founded in Fourier optics^38^. In particular, the excitation source in TFMPEM can be viewed as the superposition of numerous monochromatic waves. In the time domain, the intensity distribution of the ultrashort pulse, *I*_*G*_, on the DMD is related to the inverse Fourier transform of the complex amplitude of its spectral profile, *U*_*G*_, i.e.,

$${I}_{G}\left({x}_{G},{y}_{G},t\right)={\left|\frac{1}{2\pi }\int {U}_{G}\left({x}_{G},{y}_{G},\omega \right){e}^{j\omega t}d\omega \right|}^{2},$$

where *ω* is the angular frequency of the laser beam. When the pulse is reflected from the grating, the superposed frequencies are separated into different diffraction angles, *θ*_*ω*_, in accordance with the diffraction equation, i.e.,

$$\frac{\omega }{c}sin {\theta }_{\omega }=\frac{\omega }{c}sin {\theta }_{i}\pm m\frac{2\pi }{\Lambda },$$

where *θ*_*i*_ is the incident angle; *c* is the speed of light in vacuum; and *m* and Λ are the diffraction order and period of the DMD, respectively. The diffracted frequencies are focused independently at different points on the back focal plane of the objective lens and form a spectral line. The complex amplitude distribution of the frequency, *U*_*BF*_, which is restricted by the circular aperture of the objective lens, can be calculated in the spatial domain via Fourier transformation as.

$${U}_{BF}\left(u,v,\omega \right)=\left[\iint {U}_{G}\left({x}_{G},{y}_{G},\omega \right){e}^{-j\frac{\omega }{c}\left(\frac{u-{f}_{1}tan {\theta }_{\omega } }{{f}_{1}}{x}_{G}+\frac{v}{{f}_{1}}{y}_{G}\right)}d{x}_{G}d{y}_{G}\right]circ\left(\frac{\sqrt{{u}^{2}+{v}^{2}}}{D/2}\right),$$

where *f*_*1*_ and *D* are the focal length of the collimating lens and effective diameter of the back aperture of the objective lens, respectively. According to Fourier optics, the angular spectrum at the front focal plane of the objective lens, *A*_*TF*_, which is also defined as temporal focusing plane, is equal to *U*_*BF*_. However, this methodical approach in the spatial domain yields only an estimate of the complex amplitude of the spectral profile in the temporal focusing plane. Thus, in order to obtain a more accurate evaluation of the angular spectrum at the defocusing plane, *A*_*dTF*_, the present study utilizes the Helmholtz equation to evaluate the propagation over a distance Δ*z* (i.e., the defocusing length away from the temporal focusing plane), with the relative phase term. In other words, the angular spectrum is modeled as.

$${A}_{dTF}\left(u,v,\omega \right)={U}_{BF}\left(u,v,\omega \right){e}^{-j\frac{\omega }{c}\sqrt{{n}^{2}-{\left(\frac{u}{{f}_{2}}\right)}^{2}-{\left(\frac{v}{{f}_{2}}\right)}^{2}}\Delta z},$$

where *n* and *f*_*2*_ are the refractive index of the propagating medium and the focal length of the objective lens, respectively. The complex amplitude of the overall spectral profile in the spatial domain can then be obtained using the Fourier transform as:

$${U}_{dTF}\left(x,y,\Delta z,\omega \right)=\iint {A}_{dTF}\left(u,v,\omega \right){e}^{-j\frac{\omega }{c}\left(\frac{x}{{f}_{2}}u+\frac{y}{{f}_{2}}v\right)}dudv.$$

Finally, the intensity distribution of the temporal focusing region in the time domain, *I*_*dTF*_, is obtained from the time domain inverse Fourier transform of the spectral profile as follow:

$${I}_{dTF}\left(x,y,\Delta z,t\right)={\left|\frac{1}{2\pi }\int {U}_{dTF}\left(x,y,\Delta z,\omega \right){e}^{j\omega t}d\omega \right|}^{2}.$$

In other words, the time domain intensity distribution of the ultrashort pulse in TFMPEM can be calculated based on Fourier transformation in both the time domain and the spatial domain. The time-varying fluorescence intensity under a multiphoton excitation scheme, *I*_*MPEF*_, is proportional to the order of magnitude of the intensity distribution in the temporal focusing region. That is,

$${I}_{MPEF}\left(x,y,\Delta z,t\right)\propto {\left|{I}_{dTF}\left(x,y,\Delta z,t\right)\right|}^{M},$$

where *M* is the order of magnitude of the *M*-photon excitation process. The AEC of TFMPEM, *I*_*AEC*_, consists of the distribution of the overall, time-averaged fluorescence at different defocusing lengths, and can be estimated via the spatiotemporal integration of the time domain intensity distribution in the temporal focusing region as follow:

$${I}_{AEC}\left(\Delta z\right)=\iiint {I}_{MPEF}\left(x,y,\Delta z,t\right)dxdydt.$$

Hence, the numerical analysis model enables the computation of both the complex amplitude distributions at the back focal plane and the temporal focusing region, respectively, and the AEC of the structured lighting pattern.

### Hilbert–Huang transform

The HT utilizes two patterned images with the same spatial frequency but a 180° difference in phase. The background noise can thus be eliminated through subtraction. The HT reconstruction process introduces a phase shift of 90°. Therefore, the subtracted image and its HT can be superimposed to create an image without background noise. However, for high spatial frequencies of the structured light produced by the DMD, the detection signal has a square-like characteristic, which results in the formation of pattern residuals in the reconstructed image. Thus, the present study adopts the HHT to process the detection signal. In particular, before demodulating the signal, the input is decomposed into multiple IMFs by sifting process via enhanced fast empirical mode decomposition^39^. In general, the first few terms of the IMFs contain high frequency part of the input image, and the rest of the IMFs contains lower frequency part such as pattern residual and background noise. The filtered image without unwanted IMFs is built via selective reconstruction to perform traditional HT algorithm for image reconstruction.

## Data Availability

The datasets used and analyzed in the current study are available from the corresponding author on reasonable request.
